# A study for association and interaction analysis to metabolic syndrome and the *ESR1* gene on cardiovascular autonomic neuropathy in a Chinese Han population

**DOI:** 10.1186/s13098-016-0155-3

**Published:** 2016-07-22

**Authors:** Fangfang Zeng, Linuo Zhou, Zihui Tang

**Affiliations:** Department of Endocrinology and Metabolism, Shanghai Tongji Hospital, Tongji University School of Medicine, Shanghai, 200065 China; Department of Endocrinology and Metabolism, Fudan University Huashan Hospital, Shanghai, China

**Keywords:** Metabolic syndrome, Estrogen receptor alpha gene, Cardiovascular autonomic neuropathy, Interaction analysis

## Abstract

**Background:**

The aim of this study was to investigate the association and interaction of metabolic syndrome (MetS) and estrogen receptor alpha 1 (*ESR1*) gene polymorphisms on cardiovascular autonomic neuropathy (CAN).

**Methods:**

A large-scale, population-based study was conducted to analyze the interaction of MetS and *ESR1* gene polymorphisms to CAN, including a total of 1977 Chinese subjects. The most common studied single nucleotide polymorphism of *ESR1* gene-rs9340799, was genotyped. Multiple logistic regression (MLR) was performed to evaluate the interaction effect of environmental variables and gene polymorphisms. Interaction on an additive scale can be calculated by using the relative excess risk due to interaction (RERI), the proportion attributable to interaction (AP), and the synergy index (S).

**Results:**

After controlling potential confounders, MLR showed that significant association between MetS and CAN (p < 0.001). Interestingly, we found that the participants with MetS bearing the minor allele G had an increased CAN prevalence comparing those with allele A (p = 0.045), and a positive interaction was estimated by using RETI = 0.396 (95 % CI 0.262 to 0.598), AP = 0.216 (95 % CI −0.784 to 1.216) and S = 1.906 (95 % CI 0.905 to 4.015).

**Conclusion:**

The present findings suggest that MetS is significantly associated with CAN and provide evidence for the hypothesis that MetS and *ESR1* gene polymorphism (rs9340799) have interactive effects on CAN.

*ClinicalTrialsgov Identifier* NCT02461342

## Background

The autonomic nervous system plays a critical role in regulating the function of numerous systems/organs, exerting its control through a broad network of afferent and efferent small nerve fibres. Cardiovascular autonomic neuropathy (CAN) involves the autonomic nerve fibres innervating the heart and blood vessels [1], and is associated with a high risk of cardiac arrhythmias and sudden death, possibly related to silent myocardial ischemia [2]. CAN is rapidly growing in all populations worldwide, particularly in the developing world, and is a prevalent form of diabetic autonomic neuropathy [3]. The reported prevalence of CAN varies from 2.5 % to as high as 90 %, depending on the criteria used to identify CAN and the population studied [4].

The metabolic syndrome (MetS), made up of a cluster of cardiovascular risk factors including obesity, abdominal fat distribution, disorders of glucose and lipid metabolism and hypertension, increases the risk of future coronary and heart disease [5]. Hyperglycaemia and glycaemic variability play an important part in the development of autonomic neuropathy by contributing to oxidative stress that leads to neural damage [6]. Obesity and hypertension also contribute to cardiac autonomic dysfunction [7]. According to our previous study, MetS was strongly and independently associated with heart rate variability, and fasting plasma glucose and blood pressure were negatively correlated with cardiovascular autonomic function [8].

Oestrogen receptor α (ERα) is encoded by the *ESR1* gene which is located on the chromosome 6 (6q25.1). The beneficial effects of oestrogens on the cardiovascular system are mediated by the ERα, which is also found in the autonomic centres of the brain stem involved in cardiovascular regulation, and oestrogen can access autonomic preganglionic cells in the central nervous system [9]. Part of the cardiovascular protective effect of oestrogen may relate to the beneficial effect of oestrogen on cardiac autonomic function. A variety of single nucleotide polymorphisms (SNPs) of the *ESR1* gene are associated with cardiovascular disease. Of the polymorphisms identified in the *ESR1* gene, rs9340799 is one of the most widely investigated SNPs [10]. The rs9340799 GG genotype is significantly associated with higher diastolic mean blood pressure and lower heart rate [11].

Environmental changes can modify the gene effect in human complex diseases, such as CAN. Our previous studies investigated the associations between MetS and CAN, and risk factors analysis for the outcome [8]. However, little was known about the relationships of MetS and *ESR1* gene on CAN in Chinese Han population. In the present study, we hypothesize that MetS may modify the association of the *ESR1* gene and CAN, meaning that MetS interacts with the *ESR1* gene to increase susceptibility to CAN. The purpose of this study is to evaluate the extent to which MetS and the *ESR1* gene are associated with CAN, and the impact of MetS interaction with the *ESR1* gene on the outcome in Chinese Han population.

## Methods

### Study population

A risk factors survey for CAN was detailed earlier [12, 13], which was carried out in a random sample of the Chinese Han population. This study was approved by the Ethics Committee of Shanghai Tongji Hospital, Shanghai, China. Participants were recruited from rural and urban communities in Shanghai. Survey participants with undiagnosed CAN, aged 30–80 years, were included in this study. Our study invited a total of 3012 subjects to a screening visit between 2011 and 2012. Written consent was obtained from all patients before the study. As we described earlier [12, 13], some subjects were excluded from the study to eliminate potential confounding factors that may have influenced their CA function. Briefly, the exclusion criteria were as follows: (1) history or findings of arrhythmia and hyperthyroidism or hypothyroidism; (2) pregnancy or lactation; (3) serious hepatic or renal dysfunctions (GFR < 30 mL/min/1.73 m^2^). Complete clinical baseline data were obtained for 2092 (69.46 %) of the participants.

### Measurement

Participants were interviewed for the documentation of medical histories, medication, and history of smoking habits. Laboratory assessment for cardiovascular disease risk factors was completed, along with standardized examination for heart rate variability (HRV). As we mentioned earlier [12, 13], all study subjects underwent a complete clinical baseline characteristics evaluation after an 8-hour fast, which included: (1) history and physical examination; (2) heart rate and blood pressure; (3) fasting serum glucose and insulin; and (4) fasting plasma lipids. Systolic and diastolic blood pressure (BP) values were recorded as the mean of two physician-obtained measurements taken from the left arm of the seated participant. The day-to-day and inter-assay coefficients of variation at the central laboratory in our hospital for all analyses were between 1 and 3 %.

HTN was defined as BP ≥ 140/90 mmHg, or a history of hypertension medication. Body mass index (BMI) was calculated with weight in kilograms divided by the square of height in meters. DM was defined by oral glucose tolerance test (OGTT) or the use of insulin or hypoglycaemic medications. MetS was diagnosed according to the updated National Cholesterol Education Program/Adult Treatment Panel III criteria (WHO Western Pacific Region obesity criteria) in individuals meeting three or more of the criteria [14].

### SNP genotyping

The genomic DNA was isolated from whole blood by proteinase K digestion followed by phenol–chloroform extraction. *ESR1* (rs9340799) in 2092 Chinese Han participants was genotyped using iPLEX (Sequenom, San Diego, CA, USA) and detected by matrix-assisted laser desorption/ionization-time of flight mass spectrometry. Of these subjects, 1977 participants with complete clinical and genotype data were available for data analysis in this study. There was a 99.9 % genotype concordance rate when duplicated samples were compared across plates.

### The study outcome

In our large-scale, population-based study, this test was applied to evaluate CA function. HRV was measured non-invasively by power spectral analysis. As we described earlier, before CA function assessment, participants were to avoid alcohol, smoking, and coffee for 24 h, in order to induce a calm and quiet state. Short-term HRV analysis was performed for all subjects using a computer-aided examination and evaluation system for spectral analysis to investigate changes in autonomic regulation. In this study, CAN was diagnosed based on at least two abnormal cardiovascular autonomic reflex test results based on short-term HRV tests [15, 16].

### Statistical analysis

Continuous variables were tested for normal distribution using the Kolmogorov–Smirnov test. Variables that were not normally distributed were log-transformed to approximate normal distribution for analysis. Results are described as mean ± SD or median, unless stated otherwise. Differences in variables between subjects with non-MetS and MetS were determined by unpaired *t* test. Between groups, differences in properties were accessed by *χ*^*2*^ analysis. Univariate logistic regression was performed to determine variables associated with CAN, and to estimate confounding factors possibly disturbing the relation of MetS and SNP to CAN. Multivariable logistic regression (MLR) was carried out to control potential confounders for determining the independent association of variables with CAN. For interaction analysis, MLR was conducted to include two main variables and their interaction item to evaluate the interaction effect. Odds ratios (OR) with 95 % confidence intervals (CI) were calculated for the relative risk of MetS and SNP with CAN.

As we mentioned earlier [17], three parameters of RERI, AP, and S were used to estimate measures of interaction on an additive scale. The 95 % CI for the three parameters were estimated as the 2.5th and 97.5th percentiles of the resulting bootstrap sampling distribution. Results were analysed using the Statistical Package for Social Sciences for Windows version 16.0 (SPSS, Chicago, IL, USA). Tests were two-sided and a p-value of <0.05 was considered significant. For interaction analysis, a p-value of <0.10 was also considered to be significant.

## Results

### Characteristics of participants

The baseline clinical characteristics of the 1977 participants are listed in Table 1. There were 1310 females and 667 males (mean age, 60.50 ± 8.71 years) in the total sample. The prevalences of HTN, DM and CAN were significantly frequent in participants with MetS (p < 0.001 for all). In addition, there were significantly higher SBP, DBP, glucose profiles, lipids profiles levels and HR in participants with MetS as compared to participants without MetS, while there were lower HDL level and HRV index values in participants with MetS.

**Table 1:** The clinical baseline characteristics of individuals

Variable	non-MetS	MetS	Entire sample	p value*
*Demographical information*				
N	1194	783	1977	
Age years	59.8 ± 8.85	61.56 ± 8.37	60.5 ± 8.71	<0.001
Gender male, %	409 (34.25 %)	258 (32.95 %)	667 (33.74 %)	0.396
Height cm	161.5 ± 7.89	161.48 ± 7.79	161.49 ± 7.85	0.942
Weight kg	60.41 ± 9.78	67.69 ± 10.5	63.3 ± 10.68	<0.001
SBP mmHg	122.61 ± 17.33	135.52 ± 18.34	127.71 ± 18.82	<0.001
DBP mmHg	77.82 ± 9.38	82.8 ± 9.38	79.79 ± 9.69	<0.001
*Medical history*, %				
Smoking yes	182 (15.24 %)	122 (15.58 %)	304 (15.38 %)	0.773
HTN yes	371 (31.07 %)	557 (71.14 %)	928 (46.94 %)	<0.001
DM yes	112 (9.39 %)	307 (39.21 %)	419 (21.2 %)	<0.001
CAN yes	173 (14.49 %)	190 (24.27 %)	363 (18.36 %)	<0.001
*Laboratory assays*				
FPG mmol/L	5.01 ± 1.27	6.19 ± 2.16	5.48 ± 1.78	<0.001
PBG mmol/L	6.63 ± 2.87	9.08 ± 4.02	7.6 ± 3.58	<0.001
FINS uml/L	6.07 ± 10.53	9.07 ± 14.08	7.26 ± 12.16	<0.001
TC mmol/L	5.31 ± 0.96	5.33 ± 1.07	5.32 ± 1	0.603
TG mmol/L	1.35 ± 0.66	2.26 ± 1.14	1.71 ± 0.99	<0.001
HDL mmol/L	1.46 ± 0.32	1.2 ± 0.26	1.36 ± 0.33	<0.001
LDL mmol/L	3.18 ± 0.76	3.21 ± 0.8	3.19 ± 0.78	0.212
SCr μmol/L	76.56 ± 28.72	79.14 ± 22.9	77.58 ± 26.59	0.003
UA μmol/L	265.96 ± 78.37	302.66 ± 87.82	280.51 ± 84.17	<0.001
*HRV indices*				
HR bpm	71.57 ± 9.92	73.96 ± 10.34	72.52 ± 10.16	<0.001
TP ms^2^	938.73 ± 728.92	788.78 ± 668.07	879.34 ± 709.16	<0.001
LF ms^2^	210.37 ± 219.54	164.72 ± 189.4	192.29 ± 209.3	<0.001
HF ms^2^	203.82 ± 241.68	155.52 ± 183.24	184.69 ± 221.63	<0.001
LF/HF	1.67 ± 2.05	1.74 ± 1.87	1.7 ± 1.98	0.307
*SNP*				
rs9340799	237 (19.97 %)	147 (19.08 %)	384 (19.62 %)	0.495

* Difference analysis between non-MetS and MetS groups

*SBP* systolic blood pressure, *DBP* diastolic blood pressure, *FPG* fasting plasma glucose, *PBG* plasma blood glucose, *FINS* fasting blood insulin, *TC* serum total cholesterol, *TG* triglyceride, *UA* uric acid, *HDL* high-density lipoprotein cholesterol, *LDL* low density lipoprotein cholesterol, *SCr* serum creatinine, *UA* uric acid, *HR* heart rate, *TP* total power of variance, *LF* low frequency, *HF* high frequency, *HTN* hypertension, *MetS* metabolic syndrome, *DM* diabetes mellitus, *CAN* cardiovascular autonomic neuropathy, *SNP* single nucleotide polymorphism

### Genotyping

The minor allele (G) frequency of rs9340799 within the *ESR1* gene was 19.97 and 19.08 % in participants with MetS and participants without MetS, respectively. There were no significant differences in minor allele (G) frequency of this SNP between the two groups (p = 0.495). The minor allele (G) frequency was 0.217, 0.306 and 0.196 in a sample of Utah residents with Northern and Western European ancestry (CEU), a sample of Han Chinese in Beijing (HCB) and the present study sample, respectively (Table 2). The genotype frequency was within the Hardy–Weinberg equilibrium (p > 0.05). The primer for rs9340799 SNP is listed in Table 2.

**Table 2:** Information of single nucleotide polymorphism of *ESR1* and its primer

SNP	Gene	CH	Allele	MAF^CEU^	MAF^HCB^	MAF^TPS^	HWE
rs9340799	*ESR1*	6	G/A	0.217	0.306	0.196	0.451
Up-stream	CATCTGAGTTCCAAATGTCC						
Down-stream	GGATGAGCATTGGTCTCTAA						

*SNP* single nucleotide polymorphism, *CH* chromosome, *MAF* ^*CEU*^ minor allele frequency in Utah residents with Northern and Western European ancestry sample, *MAF* ^*HCB*^ minor allele frequency in Han Chinese in Beijing sample, *MAF* ^*TPS*^ minor allele frequency in the present sample, *HWE* p value from Hardy–Weinberg Equilibrium test in our sample

### Univariate analysis for CAN

Univariate logistic regression models were described earlier [12]. The models were developed to include age, gender, BMI, glucose profiles, lipids profiles, renal functions, medical history and rs9340799 SNP. Independent variables of age, BMI, SBP, DBP, FPG, PBG, FINS, TG, HTN, DM and MetS were significantly associated with CAN (p < 0.05 for all). Univariate logistic regression analysis showed MetS to be significantly associated with the outcome (p < 0.001); however, no significant association of rs9340799 SNP with CAN was reported (p = 0.627).

### Multiple variables analysis for CAN

Multiple variable logistic regression to include MetS and rs9340799 SNP, controlling for potential confounding factors of age, gender, PBG, FINS and renal functions, showed significant associations between MetS and CAN (p < 0.001, OR = 1.802 and 95 % CI 1.521–2.136, Table 3). There was also no significant association between rs9340799 SNP and CAN in MLR, controlling for potential confounding factors (p = 0.893).

**Table 3:** Multiple variables analysis for metabolic syndrome and rs9340799 for cardiovascular autonomic neuropathy

Variables	*β*	S.E.	p value	*OR*	95.0 % CI
MetS	0.589	0.087	<0.001	1.802	1.521–2.136
rs9340799	−0.114	0.109	0.299	0.893	0.720–1.106

*MetS* metabolic syndrome, *BMI* body mass index, and multiple variables logistic model adjusted for age, gender, PBG, FINS and renal functions

### Interaction of MetS and SNP for CAN

**Fig. 1:**
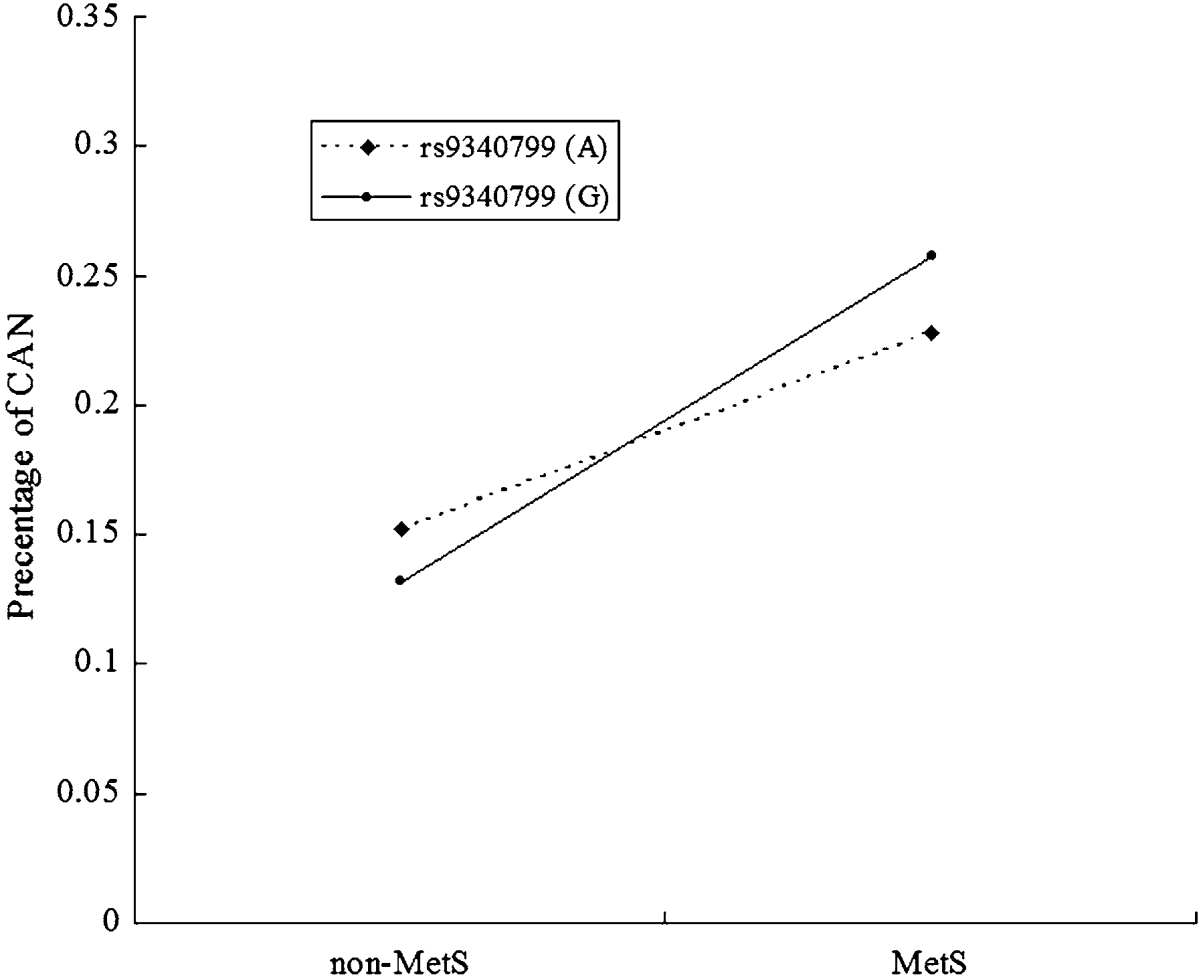
Interaction analysis of ESR1 (rs9340799) and metabolic syndrome for CAN. Interaction of MetS and rs9340799 (MetS*rs9340799 ORGEI = 1.505, 95 % CI 0.98–2.312, p = 0.062)

MLR models were developed to include the two main effect variables of MetS and rs9340799 SNP, and its interaction of MetS with rs9340799. The interaction item between them was detected in the MLR model after adjustment for relevant potential confounders involving age, gender, PBG, FINS and renal functions (p= 0.062, Table 4 and Fig. 1). In participants without MetS, the CAN prevalence was similar between subjects with minor allele G and subjects with allele A (13.39 vs. 15.60 %, p = 0.151). However, in participants with MetS, the CAN prevalence was significantly frequent in subjects with minor allele G as compared with subjects with allele A (25.59 vs. 21.89 % p = 0.045). The interaction effect was estimated as (*OR*_*Int*_ = 1.505, 95 % CI 0.980–2.312). The interaction on an additive scale was estimated as (RERI = 0.396, 95 % CI 0.262–0.598; AP = 0.216, 95 % CI −0.784 to 1.216; S = 1.906, 95 % CI 0.905–4.015).

**Table 4:** The interaction analysis of MetS and rs9340799 for CAN

Variables	*β*	S.E.	p value	*OR*	95.0 % CI
MetS	0.551	0.092	0.001	1.735	1.449–2.079
rs9340799	−0.354	0.259	0.226	0.702	0.314–1.159
MetS by rs9340799	0.409	0.219	0.062	1.505	0.980–2.312
RERI	0.396				0.262–0.598
AP	0.216				−0.784–1.216
S	1.906				0.905–4.015

*MetS* metabolic syndrome, *RERI* relative excess risk due to interaction, *AP* proportion attributable to interaction, *S* synergy index; and multiple variables logistic model adjusted for age, gender, PBG, FINS and renal functions

## Discussion

We conducted a large-scale study to evaluate the interaction effect of MetS and the *ESR1* gene on CAN in a sample of the Chinese population. To our knowledge, this is the first study to perform a synergistic analysis of MetS and rs9340799 polymorphism of the *ESR1* gene on CAN in the general Chinese population. Moreover, it is very important for us to understand environmental and genetic influences on diseases. Since more than one risk factor contributes to the progression and deterioration of a disease, finding one risk factor which is controllable, especially among modifiable risk factors, would be of great value to physicians and patients.

In the present study, there was no significant difference of allele frequency of rs9340799 between participants with and without MetS. No association of rs9340799 with CAN was detected. Univariate and multiple variable analysis provided evidence that MetS was strongly and independently associated with CAN (p < 0.001, respectively), and the results were consistent with previous studies that MetS was negatively correlated with HRV and cardiovascular autonomic function [18].

Because the incidence of coronary heart disease (CHD) rises significantly after menopause, it has been hypothesized that women’s CHD advantage before menopause, in comparison with men of the same age, could be due to the protective effects of oestrogens [19]. Additionally, oestrogen treatment, long-term or short-term, has been shown to reduce sympathetic drive and improve cardiac autonomic control [20], the same as with changes in HRV across the menstrual cycle [21]. The cardiac autonomic protective effect of oestrogen may be a partial factor in the undefined physiological mechanisms of beneficial effects of oestrogen on cardiovascular disease.

Previous studies have suggested that polymorphism rs9340799, one of the widely identified polymorphisms of *ESR1*, may relate to ischemic heart disease [22, 23]. As for the association of polymorphism rs9340799 with cardiovascular autonomic function, Matsunaga and his colleagues recruited 252 young healthy males to examine the association of *ESR1* polymorphism rs9340799 with short-term HRV [11], and found that rs9340799 GG genotype was significantly associated with higher diastolic and mean blood pressure, but lower heart rate. In our present study, no association was found between polymorphism rs9340799 and HRV indices (data not shown).

Our important finding was that a significant interaction of MetS and rs9340799 is associated with CAN in the Chinese population. In the MLR analysis, the interaction term was indicated as a significant positive interaction of the two main factors that affect CAN (p = 0.062, RETI > 0, AP > 0, S > 1), suggesting that the combined effect of MetS and rs9340799 on CAN was greater than the sum of the individual effects of the two factors. It is noteworthy that MetS individuals bearing the minor G allele were more susceptible to the progression of CAN. To our knowledge, this is the first analysis of the interaction of MetS and *ESR1* variant on CAN susceptibility. Our findings suggested that MetS interact with ESR1 polymorphisms to affect progression of CAN. In clinical practice, we may predict MetS patients with ESR1 polymorphisms more susceptibility to associate with CAN. The analysis for ESR1 polymorphisms and MetS to predict progression of CAN will be perform in follow-up studies. However, the underlying mechanism is still unknown. In the insulin-resistance atherosclerosis family study, rs9340799 was found to be positively associated with MetS [24], implying that *ESR1* (rs9340799) may disturb CAN susceptibility through unknown mechanisms contributing to MetS, which is independently and significantly associated with CAN [25]. Additionally, the indices of MetS such blood pressure and glucose profiles might modify effects on the interaction, or separately associate with the polymorphism.

Adipocyte hyperplasia and hypertrophy, insulin resistance and glucose intolerance were associated with ERα absence in the knock-out mice model, suggesting that the oestrogen/ERα signalling pathway is critical in adipose tissue and may be involved in a mechanism of energy metabolism [26]. A human male with an *ESR1*-null mutation had insulin resistance, impaired glucose tolerance, obesity and increased height [27]. The rs9340799 mutation was associated with body mass index in an age-adjusted case–control study [28], and the rs9340799 AA genotype was associated with lower body mass index and waist in middle-aged women [29]. In contrast, the rs9340799 GG genotype was associated with significantly higher serum total cholesterol and LDL cholesterol levels compared to those with the AA or AG genotype [24]. No association was found between the genotype of rs9340799 and lipid profile in our present study (data not shown). Whether polymorphism rs9340799 of the *ESR1* gene could influence serum lipid levels and the underlying molecular mechanisms still needs to be determined.

Several limitations of this study warrant comment. This study was based on a cross-sectional study; synergistic effects analysis requires a larger sample size, covering age groups other than 30–80 years and more geographic representations of China. The synergistic results in the present study need to be verified by future follow-up studies. It is also important that the study was performed on Chinese individuals and our findings may not be relevant to people of other ethnicities.

In conclusion, our findings indicate that MetS is significantly associated with CAN, and we offer evidence to support the hypothesis that MetS and *ESR1* gene polymorphism (rs9340799) have positive interactive effects on CAN. Abnormal metabolic status may modify the effect of the *ESR1* gene on the progression of CAN. This indicates that metabolic improvement of genetically high-risk individuals could attenuate the risk and progression of CAN.
